# Staghorn Calculus: A Stone out of Proportion to Pain

**DOI:** 10.5811/cpcem.2021.4.50360

**Published:** 2021-07-27

**Authors:** John Malone, Riley Gebner, Jonathan Weyand

**Affiliations:** * Madigan Army Medical Center, Department of Internal Medicine, Tacoma, Washington; † Madigan Army Medical Center, Department of Emergency Medicine, Tacoma, Washington

**Keywords:** Staghorn, infection stone, struvite, nephrolithiasis

## Abstract

**Case Presentation:**

A 25-year-old woman presented to the emergency department with two weeks of crampy right-flank pain, and urinary urgency and frequency. She was found to have a staghorn calculus filling her entire right renal pelvis on computed tomography imaging.

**Discussion:**

In contrast to ureteral calculi, staghorn calculi are more commonly observed in female patients and typically present with an indolent clinical course. A low threshold for imaging should be maintained, as prompt referral to urology for stone removal or treatment is necessary. Staghorn calculi have a high likelihood of leading to renal failure or urosepsis without treatment.

## CASE PRESENTATION

**Image 1:**
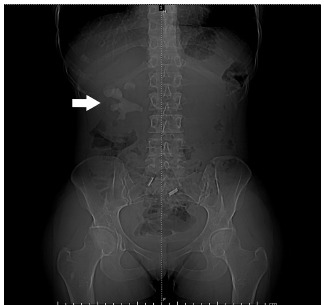
Computed tomography scout image demonstrating large staghorn calculus (arrow).

**Image 2:**
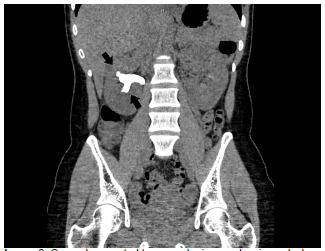
Coronal computed tomography image showing calculus filling entirety of right renal pelvis (arrows).

A 25-year-old Hispanic female presented to the emergency department (ED) with two weeks of waxing and waning right-sided flank pain. She described the pain as a cramping discomfort that worsened over the two weeks and was only mildly relieved by acetaminophen. She also reported urinary frequency and urgency without dysuria or hematuria. Physical examination was notable for right upper quadrant and mild right costovertebral angle tenderness. Urinalysis showed nitrite negative, leukocyte esterase positive urine with 685 white blood cells per high power field, 53 red blood cells per high power field, and appreciable bacteria. A computed tomography (CT) from the ED revealed a right staghorn calculus with hydronephrosis along with left nephrolithiasis (Images 1, 2).

## DISCUSSION

Staghorn calculi are the only type of renal stones more commonly observed in female patients as a result of their association with urinary tract infections.^1^ Other patient characteristics associated with struvite stones include gross hematuria, lower urinary tract symptoms, fever on presentation, a past medical history of hypertension, and multiple stones on imaging.^1^ In contrast to nephroliths in the ureters, staghorn calculi often have an insidious course with mild or no pain; therefore, we suggest a low threshold for imaging (computed tomography or ultrasound) to accelerate definitive treatment.^2^ The goal of treatment is complete removal of the stone, as any remaining fragments may harbor bacteria that are difficult to sterilize with antibiotics.^3^,^4^ Without treatment, a staghorn calculus is likely to cause renal failure, urosepsis, or both.^4^,^5^ Early recognition and referral to urology is crucial to reduce the risk of morbidity and mortality in these patients.

**CPC-EM Capsule**

What do we already know about this clinical entity?

*Struvite calculi are composed of magnesium ammonium phosphate (struvite) and calcium carbonate-apatite and are caused by urinary tract pathogens*.

What is the major impact of the image(s)?

*This image depicts struvite calculi within the renal pelvis and calyces giving the characteristic “staghorn” formation for which these stones are also named*.

How might this improve emergency medicine practice?

*As these stones are notably more insidious in presentation, earlier recognition and urgent referral will hopefully result in better outcomes for patients*.
